# The IPTA Nashville Consensus Conference on Post‐Transplant Lymphoproliferative Disorders After Solid Organ Transplantation in Children: Updates on Classification and Prevention—2026

**DOI:** 10.1111/petr.70445

**Published:** 2026-09-10

**Authors:** Michael Green, Marian G. Michaels, Thomas Gross, Upton Allen, Catherine M. Bollard, Olivia Martinez, Masaki Yamada, Steven H. Swerdlow

**Affiliations:** ^1^ Department of Pediatrics and Surgery, UPMC Children's Hospital of Pittsburgh University of Pittsburgh School of Medicine Pittsburgh Pennsylvania USA; ^2^ Phoenix Children's Hospital Phoenix Arizona USA; ^3^ Division of Infectious Diseases and the Transplant and Regenerative Medicine Center, Department of Paediatrics, Hospital for Sick Children University of Toronto Toronto Ontario Canada; ^4^ Children's National Hospital and the George Washington University Washington DC USA; ^5^ Department of Surgery and Program in Immunology Stanford University School of Medicine Stanford California USA; ^6^ Division of Infectious Diseases, Department of Medical Subspecialties National Center for Child Health and Development Tokyo Japan; ^7^ Department of Pathology University of Pittsburgh School of Medicine Pittsburgh Pennsylvania USA

## Abstract

The 2019 International Pediatric Transplant Association (IPTA) consensus conference assessed available evidence and developed recommendations for various aspects of care relating to post‐transplant lymphoproliferative disorders (PTLD) after solid organ transplantation in children. Because of the key role Epstein‐Barr Virus (EBV) plays in the pathogenesis of many PTLD cases, it was addressed in many recommendations from the Consensus Conference. Working groups addressed and published three separate manuscripts providing recommendations and identifying key knowledge gaps relating to the diagnosis, prevention, and management of PTLD and EBV infection in this setting. The definition and classification of PTLD were actively discussed, but recommendations were not addressed in these publications. In 2025, a working group of experts in EBV and PTLD initiated an effort to review, describe, and consider the impact of newer data published since the consensus conference on the appropriateness of recommendations made and the status of knowledge gaps identified through the 2019 consensus conference's efforts. This manuscript addresses the results of this work relating to the classification and prevention of PTLD and EBV in pediatric SOT.

**Trial Registration:** ClinicalTrials.gov identifier: NCT05164094; ID NCT06741072

**Abbreviations**
CMV: cytomegalovirus
CTLs: cytotoxic T lymphocytes
EBV: Epstein‐Barr virus
Ig: immune globulin
IPTA: International Pediatric Transplant Association
IVIG: intravenous immune globulin
NAT: nucleic acid amplification test
PCR: polymerase chain reaction
PTLD: post‐transplant lymphoproliferative disorder
RIS: reduction of immune suppression
SOT: solid organ transplantation
VST: virus‐specific T‐cell therapy

## 1 Background

In 2019, the International Pediatric Transplant Association (IPTA) convened an expert consensus conference to assess current evidence and develop recommendations for various aspects of care relating to post‐transplant lymphoproliferative disorders (PTLD) after solid organ transplantation in children. Epstein‐Barr Virus (EBV) plays an important role in the pathogenesis of many PTLD cases and hence was addressed in many recommendations from the IPTA Consensus Conference.

Each member of the Consensus Conference worked in one or two of the four working groups, addressing the topics of classification, diagnostics, prevention, and management of PTLD and EBV infection in this setting. Four separate reports were published, describing the methods used to complete this work, as well as reporting recommendations addressing diagnostics, prevention, and management of EBV and PTLD [1, 2, 3, 4]. The recommendations relating to the definition and classification of PTLD were never published.

In 2025, a group was organized, and a decision was made to work together to review and describe relevant data available after the 2019 consensus conference and consider the potential impact of this newer data on the recommendations made and knowledge gaps identified by the consensus conference. Invited participants included seven members of the IPTA EBV/PTLD Consensus Conference and one additional individual (MY).

## 2 Methodology

Four working groups (covering classification, diagnostics, prevention, and management) were identified to review the literature available since the consensus conference in 2019. Working groups were charged with focusing their literature reviews on the identification of new data that might be relevant to the consensus conference's recommendations or address key knowledge gaps identified in their published reports. This manuscript includes the work of the classification and prevention working groups. The goal of the former group was to provide an overview of the current status of PTLD classification, including areas of controversy and ongoing knowledge gaps. The goal of the latter group was to identify and then consider how recent published data impact recommendations and knowledge gaps addressing the prevention of EBV‐associated PTLD.

For the prevention update, literature reviews were conducted using combinations of keywords (e.g., EBV, organ transplant, each of the individual types of organ transplant (including kidney and renal transplant), prevention, prophylaxis, pre‐emptive therapy, ganciclovir, valganciclovir, acyclovir, rituximab, and heart transplant) relevant to each of the existing recommendations from the prevention working group manuscript to identify articles published after the 2019 IPTA EBV Consensus Conference. Individual papers were reviewed for relevance and inclusion. The references from these publications were also reviewed to identify additional manuscripts with relevant data. Additionally, for the vaccine section, we also searched ClinicalTrials.gov using the terms EBV and vaccines to identify vaccine trials that have been listed since the publication of the IPTA Consensus Conference guidelines. Only fully published manuscripts were used as part of our reconsideration of existing recommendations, though one published abstract describing preliminary results from one of the vaccine trials was included in our reference list for the interested reader.

A consensus from all authors was sought regarding our decision to support or modify current recommendations based on the availability of newer data. All authors reviewed the conclusions of the working groups and could provide input prior to their publication.

## 3 Summary of Status of Recommendations

### 3.1 Diagnosis and Classification of Post‐Transplant Lymphoproliferative Disorders

The basic diagnosis of PTLDs, including many of the more problematic areas, has not changed significantly since the 2019 International Pediatric Transplant Association (IPTA) consensus conference on pediatric PTLDs, in spite of greater understanding of some of their underlying biology [5, 6, 7]. The classification of PTLD, however, has continued to evolve. Although the consensus conference used the widely accepted 2016/2017 revised fourth edition WHO classification [8, 9], currently there are two different classifications in use. The 2022 International Consensus Classification (ICC) was developed by the Society for Hematopathology (SH) and the European Association for Haematopathology (EA4HP) after the 2021 Clinical Advisory Committee (CAC) meeting, following the same process used for the third, fourth, and revised fourth editions of the WHO classifications (Table 1) [10, 11]. The CAC is an opportunity for hematopathologists to get the all‐important input from hematologists, oncologists, and scientists in order to develop a consensus for classification that is both biologically meaningful and clinically relevant.

**TABLE 1:** Immunodeficiency‐associated lymphoproliferative disorders in the ICC (adapted from reference [10]).

Immunodeficiency‐associated lymphoproliferative disorders
Posttransplant lymphoproliferative disorders (PTLD)
Nondestructive PTLDs
Plasmacytic hyperplasia PTLD
Infectious mononucleosis PTLD
Florid follicular hyperplasia PTLD
Polymorphic PTLD
Monomorphic PTLD (B‐cell and T‐cell/NK‐cell types)^a^
Classic Hodgkin lymphoma PTLD^a^
Other iatrogenic immunodeficiency‐associated lymphoproliferative disorders

^a^ Further classified according to the lymphoma to which they correspond.

In contrast, the authors of the revised fifth edition WHO classification (WHO5) chose to follow the proposal of a 2015 SH/EA4HP slide workshop, as published in 2017 and 2018 [12, 13, 14]. The WHO5 rejected PTLD as a diagnostic category. It grouped all of the immunodeficiency and immune dysregulation (IDD)‐associated lymphoproliferative disorders together, including those arising in patients with HIV (human immunodeficiency virus), primary immunodeficiencies, and post‐transplant settings, under the category of “lymphoid proliferations and lymphomas associated with immune deficiency and dysregulation” and placed that diagnostic header under the umbrella of mature B‐cell neoplasms (Table 2). The WHO5 also notes that outside of inborn errors of immunity, there are too few T‐cell neoplasms in the setting of IDD to provide “convincing epidemiological evidence of a causal relationship.” [14]. The 2016/2017 WHO and ICC classifications include monomorphic PTLD of T or NK cell origin.

**TABLE 2:** Lymphoid proliferations and lymphomas associated with immune deficiency and dysregulation listed in the WHO5 (adapted from reference [14]).

*Lymphoid proliferations and lymphomas associated with immune deficiency and dysregulation*.
Hyperplasias arising in immune deficiency/dysregulation
Polymorphic lymphoproliferative disorders arising in immune deficiency/dysregulation
EBV‐positive mucocutaneous ulcer
Lymphomas arising in immune deficiency/dysregulation
Inborn error of immunity‐associated lymphoid proliferations and lymphomas

As explained further below, the IDD‐associated LPDs in WHO5 are then to be further designated using a three‐part nomenclature (Table 3) [14, 15]. The WHO5 specifically, for example, does not recommend the use of “polymorphic post‐transplant lymphoproliferative disorder” or “monomorphic post‐transplant lymphoproliferative disorder” as diagnostic terms, but in its latest iteration states that “in the posttransplant setting only, it is acceptable to include “post‐transplant lymphoproliferative disorder (PTLD)” as an addendum” [14].

**TABLE 3:** Reporting of immune deficiency/dysregulation‐associated LPDs using the 3 part nomenclature of the WHO5 (adapted from reference [15]).

Histologic diagnosis	Viral association	IDD setting
Hyperplasia (specify type)	EBV +/−	Inborn error of immunity (specify type)
Polymorphic LPD	HHV8 +/−	HIV infection
Mucocutaneous ulcer		Posttransplant (specify: solid organ/BM)
Lymphoma (classify as for immunocompetent patients)		Autoimmune disease
		Iatrogenic/therapy‐related (specify)
		Immune senescence

Abbreviations: BM, bone marrow; EBV, Epstein‐Barr virus; HHV8, human herpesvirus‐8; HIV, human immunodeficiency virus; LPD, lymphoproliferative disorder.

Although this proposal for eliminating PTLD as a diagnostic category was known at the time, it was rejected by the ICC “based in part on major differences in clinical management” [10]. In a recent Blood commentary on a PTLD treatment trial, Choquet noted that “Although this grouping [of LPD in immunocompromised patients by the new WHO] seems logical to the pathologist, this is not the case for clinicians.” [16]. Amengual and Pro, in their 2023 publication in Blood on how to treat PTLD also used the ICC [17]. In addition to very different therapeutic approaches between, for example, PTLD and LPDs arising in patients with HIV or primary immunodeficiencies, it is important to remember that neither clinical investigations nor biologic studies of PTLD can necessarily be generalized to all IDD‐associated LPDs. In fact, while there are some overlapping features among LPDs occurring in varied settings of immunodeficiency, significant differences are reported as well; however, this topic is beyond the scope of this update. Furthermore, from a very pragmatic point of view, while subcategorization of some PTLDs can be very difficult, subjective, and non‐uniform among pathologists, often those cases can still easily be classified as a PTLD, and patients can be given appropriate therapy. The updates that follow in this and subsequent diagnostic and management publications are based specifically on studies of PTLD, as defined by the 2016/2017 WHO classification and now as defined by the ICC. The conclusions are not intended to apply to other settings of immunodeficiency or immune dysregulation.

#### 3.1.1 ICC Approach to PTLD Classification

Classification of PTLD in the current ICC is essentially the same as that in the 2016/2017 WHO classification and that used in the IPTA consensus conference (Table 1) [10]. Originally published in 2022, the ICC has been explained in greater detail in the 2025 Jaffe et al. ICC‐based Hematopathology text [5, 18] and in the Arber et al. 2025 ICC text [11]. The chapter on PTLDs in the latter text does suggest minimal modifications [7]. EBV‐positive mucocutaneous ulcer (MCU) is listed as a type of PTLD, in addition to its primary listing in the ICC, where it is recognized as occurring in varied circumstances associated with immunosuppression/immunosenescence. The 2016/2017 WHO classification did recognize that EBV+ MCU, although commonly associated with advanced age, could occur following transplantation [8], and the Jaffe et al. text notes that some PTLD would fulfill the criteria for EBV+ MCU [5]. The ICC text also suggests that classic Hodgkin lymphoma PTLD should be included among the monomorphic PTLDs [7].

#### 3.1.2 Current WHO Approach to LPDs That Arise Following Transplantation

As noted above, the WHO5 approach to post‐transplantation LPDs is the same as that for LPDs occurring in varied settings of immunodeficiency/dysregulation and very different from the ICC approach. Lymphoid proliferations occurring in the setting of immunodeficiency or immune dysregulation are to be reported using a three‐part nomenclature in which the “name of the histological lesion” is provided, “the presence or absence of one or more oncogenic viruses” is indicated, and “the clinical setting/immunodeficiency background” is given (see Table 3 for details).

#### 3.1.3 Updates on How the Classification of PTLDs Impacts Clinical Management and Therapeutic Strategies in 2026 and Remaining Questions

The very heterogeneous nature of PTLD and lack of a complete understanding of its varied biology, along with the other factors discussed above, continue to make a meaningful but also clinically significant categorization that can be applied in a uniform fashion difficult. Thus, although many clinicians believe/hope that the pathologist can provide the answer as to how best to treat any particular patient based on our current classification, this remains problematic. It is well known in PTLD that lesions at different anatomic sites may have different pathologic features. Additionally, lesions may look similar pathologically; however, the best therapy for an individual patient depends on other clinical features, such as extent of disease, immunocompetency of the patient, function and risk of rejection of the allograft, and so forth. As the ability to delineate specific biology/pathogenesis of the various clinical and histologic presentations of PTLD improves, the hope is that this will translate into improved diagnosis and treatment for specific patients.

#### 3.1.4 Areas Yet To Be Resolved/Major Gaps

While a host of questions in terms of the diagnosis and classification of PTLDs remain (Table 4), the biggest issue at the present time is which basic classification approach to use—that used by the WHO classifications up to the most recent one and by the ICC, or the 2022 WHO classification and its subsequent iteration in the WHO 5th edition classification of tumors publication. An attempt is currently underway to have a universally accepted single classification of myeloid and lymphoid neoplasms, including the PTLDs [19]. The first major step in the process was a Classification Advancement Meeting, similar to the CAC of the past, planned by the SH and EA4HP, and held in March 2026. Conclusions will be published and then are to be considered by the editorial board for the next edition of the IARC published sixth edition of the WHO classification.

**TABLE 4:** Unresolved questions about PTLD diagnosis and classification.

What approach should be used for classification of PTLDs, i.e., one like the ICC, where PTLDs are considered separate from other LPDs occurring in the setting of IDD, or one like the current WHO in which the IDD‐associated LPDs are grouped, a three‐part nomenclature is used, and the diagnosis of PTLD is not recommended? Can more precise and uniformly agreed‐upon criteria for polymorphic PTLD be established? Can EBV latency patterns aid in the classification of PTLD? What should the minimum criteria be for PTLD? Can nondestructive PTLD occur at non‐lymphoid sites? Is the diagnosis of infectious mononucleosis in a transplant patient different from that of infectious mononucleosis PTLD? Are there any situations where the diagnosis of an EBV‐negative, nondestructive PTLD can be made? Can EBV‐negative marginal zone lymphomas occurring posttransplantation be considered a type of PTLD, like EBV+ MALT lymphomas are? Should EBV+ MCU be listed separately as a type of PTLD (in addition to being listed separately in the overall classification) and, if so, should it be diagnosed as EBV+ MCU PTLD? Should CHL PTLD be separately listed under PTLD or included as a type of monomorphic PTLD? With increasing numbers of ancillary studies now widely available, what is the recommended workup for suspected and definite PTLDs? Can a fine‐needle aspiration biopsy ever be sufficient for diagnosis and classification of a PTLD? What are the most important clinical and biological features underlying the development and progression of the varied types of PTLD and what are their implications for optimal therapeutic approaches? What is the relationship between the different types of PTLD and LPDs arising in immunocompetent hosts and in hosts with various types of immunodeficiency or immune dysregulation?

Many of the other issues to be resolved remain from the time of the IPTA consensus conference. Of great importance, more objective and uniformly agreed‐upon biologically meaningful criteria for polymorphic PTLD are needed, including a better definition of its borders, with some polymorphic PTLD overlapping what some consider infectious mononucleosis PTLD and others fulfilling criteria that some would consider monomorphic PTLD. It may be that something more than heavy reliance on histologic criteria will be critical or that the basic approach to the subcategorization of PTLD (which is really decades old [20]) should undergo a significant alteration. Whether assessing EBV latency patterns may be helpful in this regard is an open question. The minimal criteria for PTLD remain uncertain, given that not all lymphoid proliferations in transplant patients are PTLD and some EBV+ cells can be seen outside of diagnostic PTLDs. Can the diagnosis of a non‐destructive PTLD be made in non‐lymphoid sites, or are those types of lesions better classified as inflammatory processes, such as EBV hepatitis or EBV enteritis? Is the diagnosis of infectious mononucleosis in a transplant patient different from infectious mononucleosis PTLD? Do EBV negative non‐destructive PTLD exist? Are we ready to accept EBV negative marginal zone lymphomas as a type of PTLD, as has been previously proposed? [21]. As noted above, it has become apparent that discussions are needed as to the precise way EBV+ MCU in the transplant setting should be handled and whether classic Hodgkin lymphoma (CHL) PTLD should be included as one of the monomorphic PTLD or listed separately.

The extent of the required diagnostic workup of biopsies remains a topic of discussion. For example, should molecular/cytogenetic studies be performed routinely in biopsies with suspected or definite PTLDs to assess for chromosomal abnormalities and mutational status, or are these studies only necessary in more specific circumstances, and if so, which? In what circumstances, if any, is a fine‐needle aspiration sufficient to diagnose and classify a PTLD? Finally, in spite of progress, a complete understanding of PTLD is yet to be achieved, in part because of its great heterogeneity, the different types of organs transplanted, and varied immunosuppressive approaches. Hence, studies of the underlying biology of PTLDs in different settings and their relationship to lymphoid proliferations occurring both in immunocompetent hosts and in patients with other types of IDD need to be further investigated. The clinical implications that follow the resolution of the above questions will also need to be determined.

### 3.2 Post Transplant Lymphoproliferative Disorders: Prevention

Strategies aimed at preventing PTLD in pediatric SOT recipients have focused on the prevention or control of EBV infection to prevent the development of EBV‐associated PTLD (EBV/PTLD). The IPTA consensus conference considered available data and provided recommendations relating to both prophylactic and preemptive approaches to the prevention of EBV/PTLD [2]. A summary of the IPTA Consensus Conference recommendations and key knowledge gaps relating to the prevention of EBV/PTLD are shown in Table 5. A discussion of new data and its potential impact on these recommendations and knowledge gaps for the various prophylactic and preemptive approaches follows and is also summarized in the table.

**TABLE 5:** Updated consensus recommendations and knowledge gap for prevention of pediatric EBV‐PTLD.

Q#	Clinical question	Summary of recommendations	Evidence grade	Reaffirmation status/guideline identified key gaps and comments
1	Is there a current role for EBV vaccination in the prevention of EBV infection and disease/PTLD in pediatric SOT recipients?	Studies to date have failed to show a benefit of EBV vaccines in immunecompetent or SOT recipients, and there are no approved EBV vaccines for any population, including children undergoing SOT. Accordingly, their use cannot be recommended at this time.	Strong‐High	Reaffirmed. Addressing key gap: Studies using multiple vaccine strategies are underway in immunocompetent participants.
2	Is there evidence that the use of passive immunization with IVIG can prevent EBV disease and PTLD in previously EBV‐naïve pediatric SOT recipients?	Based on currently available data, the use of immune globulin products is not recommended for the prevention of EBV infection or disease in children undergoing SOT.	Weak‐Moderate	Reaffirmed. No additional gaps in knowledge.
3	Should monoclonal antibodies against CD20 be used prophylactically at, or soon after, transplantation in pediatric SOT recipients at increased risk of developing EBV disease and PTLD?	Use of anti‐CD20 antibodies for the prevention of EBV transmission and/or EBV disease after transplant is not recommended.	Strong‐Low	Reaffirmed. Key gap remains the need for studies evaluating prophylactic role of anti‐CD20 agents such as rituximab in pediatric subjects at higher risk for EBV‐associated PTLD (lungs and intestine).
4a	Is there a role for post‐transplant antiviral therapy (e.g., acyclovir, valacyclovir, ganciclovir, or valganciclovir) in the prevention of primary EBV infection in EBV‐naïve organ transplant recipients?	Use of acyclovir, ganciclovir, and their prodrugs is not recommended as chemoprophylaxis to prevent EBV infection in the EBV‐naïve organ transplant recipient.	Weak‐Moderate	Reaffirmed. Key gap remains the need for prospective RCT addressing antiviral vs. no antiviral to prevent EBV infection
4b	Is there a role for post‐transplant antiviral therapy (e.g., acyclovir, valacyclovir, ganciclovir, or valganciclovir) in the prevention of EBV disease (including PTLD) in EBV‐naïve organ transplant recipients?	Use of acyclovir, ganciclovir, and their prodrugs is not recommended as chemoprophylaxis to prevent EBV disease (including PTLD) in the EBV‐naïve organ transplant recipient.	Strong‐Moderate	Reaffirmed. Key gap remains the need for prospective RCT addressing antiviral vs. no antiviral to prevent EBV disease (including PTLD)
5	Who should be a candidate for EBV viral load monitoring to inform a preemptive intervention?	EBV surveillance monitoring should be performed in all EBV D+/R‐pediatric SOT recipients; it should also be considered in all EBV‐naïve children undergoing organ transplant.	Strong‐Low	Reaffirmed. No Identified gaps in knowledge
6	Can preemptive reduction of immunosuppression in response to elevated or rising EBV viral load prevent the development of EBV disease, including PTLD?	Reduction of immunosuppression in response to an elevated or rising EBV viral load is recommended to prevent the development of EBV disease, including PTLD, in pediatric liver transplant recipients. Similar reduction should be considered in other organ recipients, especially when the level of immunosuppression is deemed higher than necessary to prevent rejection in an individual with a rising EBV viral load.	Strong‐moderate for liver recipients; Weak‐Low for other organs	Reaffirmed. Key gap in available data reporting impact of preemptive reduction in immunosuppression on progression to EBV disease and PTLD for non‐liver pediatric SOT recipients with elevated EBV loads
7	Can preemptive antiviral therapy in response to an elevated or climbing EBV load in the peripheral blood prevent EBV disease and PTLD in pediatric SOT recipients?	Current data do not support the use of antiviral therapy (ganciclovir or acyclovir) as preemptive therapy to prevent development of EBV disease, including PTLD.	Weak‐Low	Reaffirmed
8	Should preemptive rituximab be used to prevent the progression of EBV DNAemia to EBV disease/PTLD in pediatric SOT recipients?	The use of rituximab as preemptive therapy to prevent the development of EBV disease, including PTLD, cannot be routinely recommended.	Weak‐Very Low	Reaffirmed. Key gap remains the need for prospective comparative trials to determine if rituximab administration, at the time of EBV DNAemia or after lack of DNAemia improvement with reduced immunosuppression, reduces the incidence of subsequent PTLD.
9	Should adoptive immunotherapy (i.e., EBV‐directed cytotoxic T‐lymphocytes) be used pre‐emptively in response to an elevated or rising EBV load to prevent EBV disease/PTLD in pediatric SOT recipients?	The preemptive use of adoptive immunotherapy (with either autologous or third party EBV‐specific T cells) is not recommended for pediatric SOT recipients at this time.	Weak‐Low	Reaffirmed. Key gap remains the need for prospective comparative trials to determine if EBV‐specific T‐cell administration, at the time of EBV DNAemia or after lack of DNAemia improvement with reduced immunosuppression, reduces the incidence of subsequent PTLD.

#### 3.2.1 Immunoprophylaxis

##### 3.2.1.1 Vaccine

Access to an effective EBV vaccine is a well‐recognized goal whose achievement has been hampered by substantial hurdles. The IPTA PTLD Consensus Conference endorsed the need for EBV vaccine trials and encouraged exploration of multiple different candidate vaccine strategies. It is notable that substantial progress has been made towards this goal, as a number of different EBV vaccine candidates, using different vaccine strategies, have progressed to the level of clinical trials in humans. Seven EBV vaccine studies currently listed on ClinicalTrials.gov as either being open or preparing to open for enrollment are summarized in Table 6 [22, 23, 24, 25, 26, 27, 28]. Three studies are particularly noteworthy, including two that have achieved enrollment goals (with preliminary results available from one of these) and a third that includes enrollment of EBV‐seronegative transplant candidates and hopes to begin recruitment in 2026. While having multiple viable EBV vaccine candidates in clinical trials is remarkable and encouraging, lessons learned from long‐standing efforts to develop a cytomegalovirus vaccine temper optimism. Should one or more of these vaccines demonstrate promising results, early implementation of trials of EBV vaccination in EBV‐naïve organ transplant candidates would seem warranted. A brief summary of these three EBV candidate vaccines follows:

**TABLE 6:** Recent EBV vaccine studies currently listed on ClinicalTrials.gov [22, 23, 24, 25, 26, 27, 28].

Study name	Sponsor	Objective	Status	Comments
A Phase 1, Randomized, Observer‐Blind, Placebo‐Controlled, 2‐Part, Dose‐Ranging Study of an EBV Candidate Vaccine, mRNA‐1195, in Healthy Participants 18 Through 55 Years of Age. ClinicalTrials.gov ID NCT05831111 [23]	Moderna	To evaluate the safety and reactogenicity of mRNA‐1195 in healthy participants.	Active, not recruiting	Location: multiple US sites. 482 enrolled. Estimated completion 10/2026. No data available
A Study of an Epstein–Barr Virus (EBV) Candidate Vaccine, mRNA‐1189, in 10‐ to 30‐Year‐Old Healthy Adolescents and Adults. ClinicalTrials.gov ID NCT05164094 [22]	Moderna	To evaluate the safety and reactogenicity of mRNA‐1189 in three predefined age groups between 10 and 30 years of age in healthy adults and healthy EBV‐seronegative adolescents	Active, not recruiting	Location: Multiple US sites. Enrollment goal achieved (*n* = 837). Analysis is ongoing with no study results available at this time. Estimated completion: 10/7/2026.
A Phase 1, Randomized, Open‐label Study to Evaluate Safety, Reactogenicity, and Immunogenicity of an Epstein–Barr Virus Candidate Vaccine, mRNA‐1189, Following Intradermal and Intramuscular Delivery in Healthy Adults 18 to 30 Years of Age. ClinicalTrials.gov ID NCT07478952 [24]	Moderna	To evaluate the safety and immunogenicity of mRNA‐1189 after intradermal and intramuscular delivery	Recruiting (as of 3/17/2026)	Location: Australia. Previously healthy participants. Estimated completion: 5/15/2027.
Safety and Immunogenicity of an Epstein–Barr Virus (EBV) gp350‐Ferritin Nanoparticle Vaccine in Healthy Adults With or Without EBV Infection. ClinicalTrials.gov NCT0445147 [25]	NIAID/NIH	Phase 1 study to evaluate the safety and immunogenicity of a 3‐dose vaccination regimen of an adjuvanted EBV gp350‐Ferritin nanoparticle vaccine in healthy adults	Active, not recruiting. Enrollment and follow‐up completed.	Location: Bethesda, Maryland, USA. Enrollment and follow‐up completed. Forty patients enrolled. Preliminary results reported in abstract form describe evidence of mild local reactogenicity with good evidence of serologic response in both EBV seropositive and seronegative recipients. Analysis ongoing.
Trial Evaluating the Immunogenicity and Safety of an Adjuvanted Epstein–Barr Virus (EBV) Glycoprotein 350 Vaccine in EBV‐seronegative Persons. ClinicalTrials.gov ID NCT05683834 [27]	NIAID/NIH	Multisite, randomized, double‐blind Phase 1/2 study to evaluate the immunogenicity and safety of a 3‐dose regimen of an adjuvanted EBV glycoprotein 350‐Ferritin nanoparticle vaccine in EBV‐seronegative 18–25 year olds.	Active, not recruiting	Location: two sites in the USA. Recruitment completed: 25 patients. Anticipated analysis completion: 4/1/2027. No data available.
Epstein–Barr Virus (EBV) gH/gL/gp42‐Ferritin Nanoparticle Vaccine With or Without gp350‐Ferritin in Healthy Adults With or Without EBV Infection. ClinicalTrials.gov ID NCT06908096 [28]	NIAID/NIH	Phase 1 study to evaluate the safety of a 3‐dose vaccination regimen of an adjuvanted EBV gH/gL/gp42‐ferritin nanoparticle vaccine with or without gp350‐ferritin in healthy EBV‐negative or EBV‐positive people aged 18–29	Recruiting	Location: Bethesda, Maryland, USA. Estimated completion 10/1/2028. No data available.
BZLF1 Peptide Vaccine (OSU‐2131) With QS‐21 for the Prevention of Epstein–Barr Virus‐Related Cancer in Patients Awaiting Solid Organ Transplants. ClinicalTrials.gov ID NCT06741072 [26]	Ohio State University Comprehensive Cancer Center	A Phase 1B double‐blind, randomized trial evaluating the safety, tolerability, and immune response of OSU‐2131, a BZLF1 peptide vaccine with Stimulon QS‐21 solution adjuvant, in healthy volunteers and patients awaiting solid organ transplantation	Suspended—study drug issue	Location: Columbus, Ohio, USA. No information available on when study drug issue will be resolved Current estimated completion 12/31/2027

The safety and reactogenicity of the first EBV candidate vaccine, mRNA‐1189, is evaluated in a study of three predefined age cohorts in healthy volunteers 10–30 years (ECLIPSE Study, Moderna) [22]. Participants receive three doses of mRNA‐1189 or placebo. While the primary endpoints focus on safety and reactogenicity, secondary endpoints address the immunogenicity of this candidate vaccine. The study recently achieved its enrollment goal of 867 participants (ClinicalTrials.gov). Analysis is ongoing, with no study results available at this time. Two additional studies sponsored by Moderna are also evaluating mRNA‐1189 in previously healthy adults [23, 24], one of these has also completed enrollment with ongoing follow‐up of participants [23] while the other has just begun enrolling (Table 6) [24].

A study of a second vaccine candidate, EBV gp350 ferritin nanoparticle (FNP) vaccine (developed by the NIH), has also completed enrollment in a small Phase I trial evaluating safety and immunogenicity of three doses of vaccine (NCT0445147) [25]. Forty healthy individuals (20 EBV seropositive and 20 EBV seronegative individuals) were enrolled; 19 and 16 of the EBV+ and EBV‐ participants, respectively, completed the trial. Preliminary results in abstract form describe evidence of mild local reactogenicity with good evidence of serologic response in both EBV seropositive and seronegative recipients [29].

A third vaccine candidate, BZLF1 peptide vaccine (OSU‐2131) with QS‐21, is planned to be used in a study evaluating a vaccine for the prevention of EBV‐related cancer in healthy adults and patients awaiting solid organ transplants (ClinicalTrials.gov) [26]. The original intent of the study organizers was to open this study to enrollment in the spring of 2026. However, a review of study status on ClinicalTrials.gov identifies that this study has been suspended due to an issue with the study product.

##### 3.2.1.2 Prophylactic Use of Rituximab

The use of anti‐CD20 monoclonal antibody as prophylaxis against the development of EBV infection was not recommended in the IPTA guidelines (strong recommendation/low level of evidence). The absence of adequate data to define the benefits and risks of this strategy in pediatric SOT recipients was identified as a knowledge gap by the consensus conference. The guidelines noted the need for data, especially relating to prophylactic use in lung and intestine recipients, given that they are at the highest risk of developing EBV/PTLD after SOT.

Two studies published since the consensus conference report on the incidence of EBV/PTLD in patients who received rituximab as part of induction for indications unrelated to EBV. The impact of rituximab, used as part of induction, on the subsequent development of EBV/PTLD was evaluated as part of the multicenter, nationwide observational adult Swiss Transplant Cohort Study [30]. Thirty‐nine patients with biopsy‐proven EBV‐positive PTLD were identified among 4765 SOT recipients transplanted between 2008 and 2019. None of these cases developed in the 191 SOT recipients who received rituximab as part of induction. Kidney recipients with ABO‐mismatched donors accounted for the vast majority of those receiving rituximab for induction; 8% of patients in this cohort were lung transplant recipients, all of them were adults. Mean PTLD‐free survival time at 9 years of follow‐up was significantly shorter in patients not receiving rituximab than in those receiving it as part of induction treatment. Of note, none of the 121 patients receiving rituximab for treatment of rejection experienced PTLD as of the time of publication.

A second published cohort study of adult and pediatric kidney and kidney‐pancreas transplant recipients from Norway and Denmark also evaluated the impact of rituximab used as part of induction on subsequent development of EBV/PTLD [31]. The authors evaluated the hazard ratio (HR) at 2 years and 10 years post‐transplant, comparing the incidence of EBV/PTLD in EBV+ and EBV‐negative SOT recipients who received or did not receive rituximab as part of induction therapy for indications unrelated to EBV (e.g., sensitized patients). They found an HR of 0.2 (95% CI 0.3–1.49) at 2 years and 0.43 (95% CI 0.14–1.38) at 10 years post‐transplant when controlling for age (< 18 or ≥ 18 years), use of antithymocyte globulin in induction, and use of antiviral prophylaxis. While there appears to be a trend towards a protective effect of use of rituximab as part of induction, the authors point out that only 28 EBV seronegative recipients, who were at highest risk of developing EBV/PTLD, received rituximab as part of their induction, limiting the conclusions that could be drawn from this work.

Results of these two retrospective studies suggest that use of rituximab or other anti‐CD20 monoclonal antibodies used as part of induction might provide a protective effect against development of early PTLD, while their impact on late PTLD is less clear. Given the limitations of this data, continued support for not recommending the use of anti‐CD20 monoclonal antibodies as part of induction when their use is primarily to prevent the development of PTLD is appropriate. Neither study adds enough experience in lung or intestinal recipients to address the knowledge gap focusing on these patients raised by the IPTA consensus conference.

#### 3.2.2 Chemoprophylaxis

The IPTA PTLD consensus conference did not recommend the use of antiviral agents (e.g., acyclovir, ganciclovir, and their prodrugs) as chemoprophylaxis to prevent EBV infection or disease in EBV‐naïve organ transplant recipients (Table 5). These recommendations were supported by available data, which was mainly limited to retrospective, single center studies or registry‐based investigations, and were consistent with conclusions drawn by a systematic review and meta‐analysis published in 2017 by AlDabbagh and colleagues, which failed to show a benefit of acyclovir or ganciclovir‐based prophylaxis for the prevention of EBV PTLD (RR of 0.95 (95% CI 0.58–1.54)) [32]. In 2025, Moghadamnia and colleagues published a second systematic review and meta‐analysis with different conclusions. AlDabbagh found no protection for those receiving antiviral prophylaxis based on data derived from nine studies. However, Moghadamnia found evidence of protection (RR of 0.77 (95% CI 0.06–0.94)) using data extracted from 18 studies [33]. Unexpectedly, the RR for high‐risk (EBV D+/R‐) patients was 1.13 (95% CI 0.72–1.78) suggesting a lack of protective effect in this high‐risk group, raising questions as to how to interpret these divergent results.

Both meta‐analyses were designed to evaluate the protective effect of antiviral prophylaxis against the development of EBV/PTLD. Moghadamnia and colleagues included results extracted from seven studies published after the conclusion of the AlDabbagh study. While a substantial number of studies are included in both analyses, others are included in one but not the other despite being published during a period included in both meta‐analyses. Most importantly, the vast majority of studies included in both meta‐analyses are single‐center reports that retrospectively compare patients who received antivirals as prophylaxis against CMV with historical controls who did not, often as a secondary analysis in a study focused on CMV outcomes.

The limitations and quality of available data at the time of the consensus conference prompted a call for a prospective, randomized, multicenter trial(s) comparing EBV high‐risk patients (e.g., D+/R−) with and without antiviral chemoprophylaxis to definitively resolve questions about the efficacy of antiviral therapy for the prevention of EBV disease and PTLD. While the publication of Moghadamnia and colleagues' findings adds to a sense of equipoise regarding the efficacy of antiviral chemoprophylaxis for the prevention of EBV and PTLD, it does not provide convincing evidence of efficacy given the results of the AlDabbagh study. While some centers might find selective use of antiviral chemoprophylaxis in EBV‐naïve pediatric SOT recipients “at high risk” of EBV infection and PTLD attractive, we feel that the evidence continues to not support a recommendation for its use in this setting, although the potential presence of equipoise does confirm the need for new data from better designed, prospective trials.

The challenge in designing and carrying out appropriate prospective trials addressing the question of the efficacy of chemoprophylaxis against EBV infection and PTLD is doing so in the setting of having to use effective CMV prevention strategies in these children. Several potential opportunities could be considered by including this question in studies evaluating CMV prevention using preemptive therapy approaches using (val) ganciclovir or in trials evaluating the use of letermovir as CMV chemoprophylaxis in pediatric SOT recipients. Comparative studies of chemoprophylaxis and pre‐emptive therapy remain an area of interest, and as interest in letermovir use increases in the pediatric SOT population, the design and implementation of studies to address both CMV and EBV related questions would seem feasible.

#### 3.2.3 Pre‐Emptive Interventions

##### 3.2.3.1 Reduction of Immune Suppression (RIS)

The consensus conference recommended RIS in response to an elevated or rising EBV viral load to prevent the development of EBV disease, including PTLD, in pediatric liver transplant recipients. Likewise, they recommended considering the use of RIS in other organ transplant settings, especially when the risk of rejection is low (Table 5). However, they highlighted that the key knowledge gap associated with this recommendation was the paucity of published data describing the impact and safety of pre‐emptive RIS on progression to EBV disease and PTLD in children receiving non‐hepatic organ transplants. Data are needed on both which immunosuppressive agent(s) should be reduced and by how much. This is particularly important as these children are often on immunesuppression regimens containing multiple agents. Unfortunately, no studies addressing pre‐emptive reduction of immunesuppression for non‐liver recipients appeared to have been published since the IPTA guidelines were published. Until this information becomes available, the current extension of this recommendation to non‐liver organs (but with a weak recommendation and recognition of the low‐quality evidence) remains appropriate.

##### 3.2.3.2 Pre‐Emptive Use of Antivirals

The IPTA EBV PTLD guidelines noted that current data did not support the use of antiviral therapy (e.g., ganciclovir, valganciclovir, acyclovir, and valacyclovir) as preemptive therapy to prevent the development of EBV disease, including PTLD (Table 5). The consensus conference felt that available data adequately answered this question and did not identify a knowledge gap related to the preemptive use of antiviral therapy to prevent EBV disease, including PTLD. This recommendation continues to be appropriate at this time.

##### 3.2.3.3 Pre‐Emptive Use of Rituximab

The preemptive use of rituximab to prevent progression to PTLD was not recommended by the IPTA EBV/PTLD consensus conference (Table 5). This recommendation was driven by the absence of data describing the efficacy and safety of preemptive rituximab, which was identified as an important gap in knowledge that should be considered a high research priority. To best address this knowledge gap, the guidelines called for prospective comparison trials to determine whether rituximab administration, either as an initial response to EBV DNAemia or after lack of DNAemia improvement/resolution with reduced immunosuppression, reduces the incidence of subsequent PTLD.

A survey conducted prior to the 2019 IPTA PTLD Consensus Conference by the European Reference Network on Pediatric Transplantation (ERN) found that 38% of participating centers reported some use of preemptive rituximab [34]. This high level of preemptive use was not based on published efficacy data and likely did not consider the risk of or monitor for rituximab‐associated hypogammaglobulinemia, as data on this sequela were lacking at the time of the survey. More recently, a 2021 study found that 69%, 63%, and 17% of pediatric SOT recipients receiving rituximab as part of treatment for PTLD were hypogammaglobulinemic at 6 months, 2 years, and 5 years, respectively, after receiving this treatment [35]. It is likely that preemptive use of rituximab against EBV also brings the risk of hypogammaglobulinemia, though the frequency and durability of this complication in this setting have not been defined.

Unfortunately, prospective, randomized trials as proposed by the consensus conference have not been implemented to date. Published experience is limited to three retrospective case series describing preemptive use of rituximab in EBV seronegative pediatric kidney recipients experiencing primary infection who developed persistently elevated EBV loads after primary infection despite a trial of reduced immunesuppression [36, 37, 38]. The patients from the smallest of these studies were included in the larger, multicenter study [36]. Collectively, these studies included only 36 patients who received preemptive rituximab. Despite the wide use reported in the ERN study [34], additional single center or collaborative experience with preemptive rituximab does not appear to have been published.

All 36 patients who received preemptive rituximab were EBV‐seronegative prior to kidney transplant [37, 38]. Patients received one to four doses of rituximab after a trial of reduction of mycophenolate; tacrolimus doses were not reduced. All of the children experienced an initial drop in EBV load after rituximab treatment, but long‐term follow‐up was not available for all children. The collective experience demonstrates that EBV load elevations frequently recur. Two late cases of EBV/PTLD in prior recipients of rituximab were reported in the larger series by Ashoor and colleagues [38]. They also reported that 3 of 26 children developed hypogammaglobulinemia in their rituximab treated patients, but acknowledged that this rate might be higher since systematic monitoring for this complication was not included as a part of their protocol for preemptive rituximab treatment [38].

Given the continued lack of adequate data to address this knowledge gap, the need for prospective comparison trials to determine whether rituximab administration, either as an initial response to EBV DNAemia or after lack of DNAemia improvement/resolution with reduced immunosuppression, reduces the incidence of subsequent PTLD, as called for by the consensus conference, remains a research priority. Continued support for not routinely recommending the preemptive use of rituximab (or other anti‐CD20 biologics) is appropriate. The need for a well‐designed clinical trial to address this question is perhaps the most pressing research priority for the prevention of EBV/PTLD in pediatric SOT recipients.

##### 3.2.3.4 Preemptive Virus‐Specific T‐Cell Therapy (VST)

The preemptive use of adoptive immunotherapy (with either autologous or third‐party EBV‐specific T cells) was not recommended for pediatric SOT recipients at the time of the consensus conference (Table 5). However, the guidelines recognized that, if available, EBV‐specific VST might have utility and should be studied as pre‐emptive therapy, either at the time of EBV DNAemia or after lack of DNAemia improvement with reduced RIS, to assess its potential impact on the incidence of subsequent PTLD. To date, no additional studies reporting on pre‐emptive EBV VST in SOT recipients have been published, and completion of such trials would seem a lower priority than conducting the randomized trials of rituximab as preemptive therapy. Accordingly, ongoing support for the recommendation against the use of pre‐emptive EBV‐specific T cells outside of clinical trials is warranted.

#### 3.2.4 Areas Yet To Be Resolved/Major Gaps

Despite important advances in the prevention of EBV‐associated PTLD in pediatric SOT recipients, substantial gaps remain. Most prevention strategies continue to rely on low‐quality evidence derived largely from retrospective, single‐center studies, with few prospective randomized trials available to guide practice. Although several promising EBV vaccine candidates are now in clinical trials, no vaccine has yet demonstrated efficacy in preventing EBV infection or PTLD in high‐risk transplant populations.

Uncertainty persists regarding the optimal use of rituximab, both prophylactically and preemptively, including its true efficacy, long‐term safety, risk of hypogammaglobulinemia, and applicability in high‐risk groups such as lung and intestinal transplant recipients. Critical knowledge gaps also remain regarding reduction of immunosuppression, including which agents should be reduced, by how much, and in which organ transplant populations this strategy is most effective and safe.

Finally, while virus‐specific T‐cell therapies are biologically promising, evidence supporting their preemptive use is extremely limited. Collectively, these unresolved issues highlight the urgent need for multicenter prospective and randomized studies to establish evidence‐based prevention strategies for EBV/PTLD in children undergoing solid organ transplantation.

## Author Contributions

All authors contributed to the development and implementation of this Update to the findings and recommendations of the 2019 IPTA EBV/PTLD Consensus Conference. S.H.S. and T.G. led efforts relating to classification. M.G. and M.G.M. led efforts relating to prevention, O.M. and M.Y. led efforts relating to viral load and diagnostics. U.A., T.G. and C.M.B. led efforts relating to management. All authors reviewed and approved of content in this manuscript including updates to recommendations and assessments of knowledge gaps.

## Conflicts of Interest

C.M.B. is on the scientific advisory boards for Catamaran Bio and Mana Therapeutics with stock options and/or ownership, is on the Board of Directors for Caballeta Bio with stock options, and has stock in Neximmune and Torque Therapeutics. C.M.B. has patent applications related to EBV‐specific T cells licensed to Cellmedica, has served on ad hoc advisory boards for BMS, Pfizer, Roche and CDR‐Life AG and serves on the DSMB for SOBI. MG serves on a DSMB for Bristol Myers Squibb, ITB‐MED and on advisory board for ADMA. The following authors have no relevant conflicts to declare: U.A, T.G, M.G.M, M.Y, O.M, S.H.S.

## Linked Article

This article is linked to Green et al. papers. To view these articles, visit https://doi.org/10.1111/petr.14350.

## Data Availability

Data sharing not applicable to this article as no datasets were generated or analysed during the current study.
